# Presence of Specific Periodontal Pathogens in Prostate Gland Diagnosed With Chronic Inflammation and Adenocarcinoma

**DOI:** 10.7759/cureus.17742

**Published:** 2021-09-05

**Authors:** Leela Subhashini C Alluri, Andre Paes Batista da Silva, Shiv Verma, Pingfu Fu, Daniel Lee Shen, Gregory MacLennan, Sanjay Gupta, Nabil F Bissada

**Affiliations:** 1 Periodontics, Case Western Reserve University School of Dental Medicine, Cleveland, USA; 2 Periodontics, Private Practice, Oklahoma, USA; 3 Urology, Case Western Reserve University School of Medicine, Cleveland, USA; 4 Population and Quantitative Health Sciences, Case Western Reserve University School of Medicine, Cleveland, USA; 5 Pathology, University Hospitals Cleveland Medical Center, Cleveland, USA; 6 Pathology, Case Western Reserve University School of Medicine, Cleveland, USA

**Keywords:** periodontal pathogens, periodontal disease, prostate cancer, benign prostate hyperplasia, prostatitis

## Abstract

Background

Intraprostatic inflammation is frequently observed in the prostate and linked to prostatic diseases, including prostatitis, benign prostatic hyperplasia (BPH), and cancer. The etiology of prostate diseases is unclear. Periodontal diseases are associated with an increased risk of prostate diseases. In men, chronic prostatitis and moderate/severe periodontitis have significantly elevated serum prostate-specific antigen (PSA) levels. Treatment of periodontal disease reduced PSA levels in men. The presence of periodontal pathogens deoxyribonucleic acid (DNA) was identified in the prostate fluid of prostatitis patients. These pathogenic bacteria might have the potential to trigger prostatitis progressing to prostatic adenocarcinoma. The mechanism(s) explaining the etiology of association between periodontal disease and prostate cancer remains unclear. However, the presence of periodontal pathogens has not been analyzed in the prostate gland.

Objective

To identify and compare the presence of specific periodontal pathogens in the areas of BPH, inflammation, and cancer of the prostate glands diagnosed with malignancy.

Materials and methods

Whole-mount radical prostatectomy sections from men (n=30) were identified for BPH, inflammation, and cancer areas and marked for tissue procurement. The tissues were subjected to DNA isolation and analysis of microbial DNA and total bacterial load for the following pathogens, including *Porphyromonas gingivalis *strain ATCC 33277,* Prevotella intermedia *strain B422,* Treponema denticola *strain 35405, *Fusobacterium nucleatum subsp. fusiform *strain*, Tannerella forsythia *strain ATCC 43037, and *Campylobacter​​​​​​​ rectus* strain ATCC 33238performed real-time PCR. The universal bacterial primer pairs were used to detect genomic DNA (gDNA) from the total bacteria present in the samples. All species-specific primers were designed to target the variable regions of the 16S ribosomal RNA (rRNA). Data were analyzed using the 2-ΔΔCT method, statistically validated using unpaired t-test and ANOVA test.

Results

A total of 90 samples of prostate tissue specimens were analyzed for periodontal pathogens; only one pathogen (*F. nucleatum *subsp.* fusiform *strain ATCC 51190) showed a significant difference compared to the expression of *S. epidermidis *(internal control). In particular, *F. nucleatum *expression was 9, 11.9, and 10.3-fold higher in BPH, inflammation, and cancer, respectively, at p-value <0.05. Moreover, the bacterial load abundance/expression was almost similar in BPH (46.8-fold), inflammation (40.9 fold), and cancer (41.5 fold) higher. There was no significant difference in bacterial load (folder change) among the three areas of BPH, inflammation, and cancer (p-valve>0.05). Similarly, there was no significant difference between *F. nucleatum* (folder change) among the three areas (p-valve>0.05).

Conclusion

*Fusobacterium nucleatum* is identified in the prostates that harbor cancer, chronic inflammation, and BPH.

## Introduction

There are well-established associations between periodontal pathogens and various non-malignant and malignant diseases, such as cardiovascular disease, gastrointestinal disorders, adverse pregnancy outcomes, Alzheimer's disease, respiratory tract infections, diabetes, and malignant disease such as esophageal, pancreatic, colorectal, and lung cancer [1]. Studies have demonstrated that oral pathogens such as *Fusobacterium nucleatum* and *Porphyromonas​​​​​​​ gingivalis* are associated with gastrointestinal diseases and colorectal carcinoma [2]. Periodontal pathogens such as *P. gingivalis, Capnocytophaga sp., Prevotella sp., Fusobacterium sp., Streptococcus sp., *and *Peptostreptococcus* *sp.* are associated with oral cancer [2]. *P. gingivalis* and *F. nucleatum* are associated with the development of pancreatic and colorectal cancer. In pancreatic cancers, oral pathogen-associated such as *F. nucleatum, P. gingivalis, Aggregatibacter​​​​​​​ actinomycetemcomitans, Neisseria* *sp*, and *Streptococcus mitis* are reported [3-5]. In individuals with lung cancers, oral microbes such as *Capnocytophaga* and *Veillonella* are reported [6]. In esophageal cancer, oral pathogens including *Treponema​​​​​​​ denticola, Streptococcus mitis,* and *Streptococcus anginosus* are reported* *[7]. In the prostate, chronic intraprostatic inflammation and its association with periodontal pathogens have been elucidated [8]. Joshi et al. reported that individuals with both moderate/severe prostatitis and periodontitis had significantly elevated levels of prostate-specific antigen (PSA) [9]. Alwithanani et al. demonstrated that treating chronic periodontal disease in men reduced PSA levels and improved clinical symptoms of prostatitis [10]. Estemalik et al. further substantiated the association between periodontitis and prostatitis, as evidenced by the existence of the periodontal pathogens *P. gingivalis* and *T. denticola* DNA in the expressed prostatic secretion (EPS) and dental plaque of the same patient [11]. These findings provide evidence that periodontal pathogens may disseminate through the systemic circulation to initiate infection and local inflammatory response. However, the presence of periodontal pathogenic bacterial DNA has not been evaluated in the prostate gland. The present study aims to demonstrate the presence of periodontal pathogens in the prostate, which might initiate an inflammatory response.

## Materials and methods

This study is a collaborative effort between the Department of Periodontics at the CWRU School of Dental Medicine, Department of Pathology at the University Hospitals Cleveland Medical Center, and Department of Urology at CWRU School of Medicine, Cleveland, Ohio. The University Hospitals Institutional Review Board approved the protocol for the study from October 2018 to June 2021 with an IRB number 02-14-36.

Prostate gland tissue specimen’s preparation

**Figure 1:**
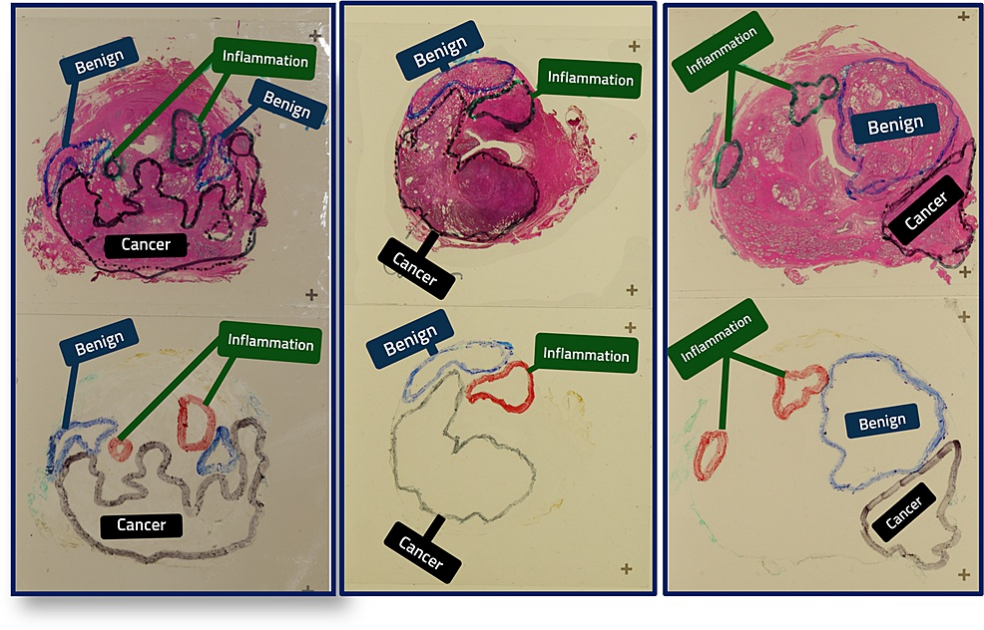
Hematoxylin and eosin-stained and unstained formalin-fixed, paraffin-embedded prostate tissue slides

Archival human prostate gland tissues from the Department of Pathology were utilized. As part of the inclusion criteria, selected radical prostatectomy specimens contained areas of benign prostatic hyperplasia (BPH), chronic inflammation, and cancer prostatic tissue; from adult male patients, including all racial backgrounds and ethnic groups. Whole-mount radical prostatectomy sections from 30 men were obtained, surgically removed with the diagnosis of prostatic adenocarcinoma. First, all case slides were reviewed by one experienced pathologist (MC) and achieved the consensus in recognizing the areas of BPH, inflammation, and cancer in the specimens. The hematoxylin and eosin (H&E) slides are given the serial number and transferred to the histology laboratory to obtain the prostate tissues that are cut into thin sections and fixed on a slide. Once the slide was prepared at the 4-µm thickness and stained with H&E, and then, ten slides of the same sample with a 10-µm thickness of unstained formalin-fixed, paraffin-embedded prostate tissue were prepared. An experienced pathologist identified and marked with three different colors: green color for the areas of BPH, red color for the inflammation, and black color for cancer on a 4-µm thickness slide. Then, using the 4-µm thickness H&E stained slide as a template marked the three areas on all the ten unstained formalin-fixed, paraffin-embedded prostate tissue slides (Figure 1).

The paraffin-embedded tissue is procured with the help of a scalpel blade, collected, and transferred to the autoclave test tubes, and given a specific serial number.

Selection of periodontal pathogens

The 16S rRNA sequence GeneBank accession number for *P. gingivalis* strain is ATCC 33277 (AF414809.1),* F. nucleatum subsp. fusiform *strain ATCC 51190 (FJ196708.1), *C. rectus* strain ATCC 33238 (JX912524.1), *T. forsythia* strain ATCC 43037 (NR_040839.1), *P. intermedia* strain B422 (NR_026119). *T. denticola* strain ATCC 35405 (NR_074582.1) was chosen in the current study.

16SrRNA extraction and quantification

The 16SrRNA was extracted from embedded paraffin tissues of the patient's sample categorized as (i) BPH (ii) inflammation, and (iii) cancer. QIA amp DNA mini kit (Qiagen USA) extracted gDNA from the above samples following the manufacturer's protocol. Following the addition of 200 µg RNase (Qiagen, USA), the samples were incubated for a further 15 minutes at 370 °C. DNA free of RNA contamination was then purified and quantified. The isolated 16SrRNA was quantified using NANO drop, considering the ratio A260/A280 in a range of 1.9-2.0. The purified and quantified DNA was further used for qPCR.

16SrRNA isolation quantification and normalization

The isolated 16SrRNA from all 90 samples, which comprised 30 patients’ samples categorized as BPH, inflammation, and cancer, were quantified and normalized to 10 ng/µl for each sample. Among the 90 samples, three samples belonging to the same patient were disregarded due to inadequate tissue samples.

Design of the primers for real-time PCR

The designed primers were taken from commercially available website-integrated DNA technology (IDT). Set parameters for primer design base-pair length of 90-100 bp were considered, and a melting temperature of between 55 and 65 °C with a GC content ranging from 35-70%.

16S rRNA quantitative polymerase chain reaction- qPCR analysis

The qPCR was performed using the samples, as mentioned above, targeting the specific region of pathogens. The reaction was performed in 12 µl reaction volume contain 2× SYBR green universal PCR master mix (Radiant™ SYBR Green, Thermo Fisher Scientific, Waltham, MA), 200 nM primers each, and 10 ng of DNA in each reaction in Applied Biosystems (7500). The thermal cycling conditions used to perform the reaction were 50 °C × 2 min, 95 °C × 10 min, 45 cycles of 15 s at 95 °C, and 45 seconds at 60 °C. For each run, at least three negative template controls (NTC) were included. For internal control, *Staphylococcus epidermidis* was used, and bacterial load was analyzed in all samples.

Data normalization and statistical analysis

The data were normalized with the expression CT values of *S. epidermidis*, and expression fold change for each sample was calculated using the 2^(- ΔΔcT) method [12]. Unpaired T-test and analysis of variance (ANOVA) were used for further statistical analysis. All tests were two-sided, and a p-value of less than 0.05 was considered statistically significant.

## Results

In the present study, patients' ages ranged from 51 to 74 years, and regarding racial background, seven patients are African Americans, 22 are Caucasians. Furthermore, the PSA level of 21 patients ranges from <0.1 to 18.80 ng/mL, and the data of eight patients' PSA levels are missing. Regarding prostate cancer tumor grade, 12 patients had a tumor grade of 3+4, nine patients had a tumor grade of 4+3, three patients had a tumor grade of 3+3, two patients had a tumor grade of 4+5, and one patient had a tumor grade of 4+4. The data of the two patients' tumor grades are missing. Additionally, in the staging of prostate cancer, ten patients had stage T3aN0; subsequently, nine patients had stage T2N0, six patients had stage T3bN0; one patient had stage T1N0, one patient had stage T2NX, one patient had stage T3aN1, and one patient had stage T3aNX (Table 1).

**Table 1:** Demographics of the patients

Age range (years)	51-74
Race	Caucasians (n=22)
	African Americans (n=7)
Total PSA range	<0.1 to 18.80 ng/mL (the PSA level of 21 patients)
	8 patient PSA levels are missing
Tumor grade	3+4 (12 patients)
	4+3 (9 patients)
	3+3 (3 patients)
	4+5 (2 patients)
	4+4 (1 patient)
	Tumor grade for 2 patients’ data not found
Tumor stage	T3aN0 (10 patients)
	T2N0 (9 patients)
	T3bN0 (6 patients)
	T3aN1 (1 patient)
	T3aNX (1 patient)
	T2NX (1 patient)
	T1N0 (1 patient)

Among the study sample, the expression of *S. epidermidis* (control) and *F. nucleatum *was compared in BPH, inflammation, and cancer. Unpaired two-tailed t-test (Table 2) and ANOVA were performed (Table 3).

**Table 2:** The difference of S. epidermidis and F. nucleatum within the areas of BPH, inflammation, and cancer examined using an unpaired T-test BPH: benign prostatic hyperplasia

	Expression	Mean	STD	p-value
Area of BPH	S. epidermidis	27.04	0.96	0.0001
	F. nucleatum	25.7	2.83	
Area of Inflammation	S. epidermidis	27.21	1.12	0.0004
	F. nucleatum	25.79	3.16	
Area of cancer	S. epidermidis	27.2	0.94	0.0371
	F. nucleatum	26.36	3.43	

**Table 3:** The difference of bacterial load and F. nucleatum among three areas examined using ANOVA

		BPH	Cancer	Inflammation	p-value
Bacterial load	Mean	46.8	41.5	40.9	0.9
	STD	59.8	51.1	49.2	
F. nucleatum	Mean	9.9	10.3	11.9	0.879
	STD	15.7	13.1	17.6	

**Figure 2:**
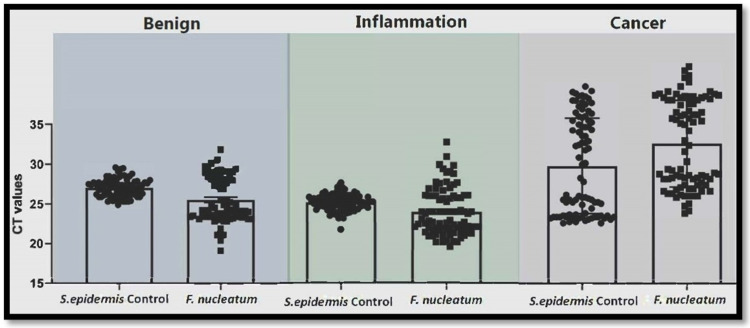
CT values of control S. epidermidis and F. nucleatum of all three areas, i.e., BPH, inflammation, and cancer BPH: benign prostatic hyperplasia

**Figure 3:**
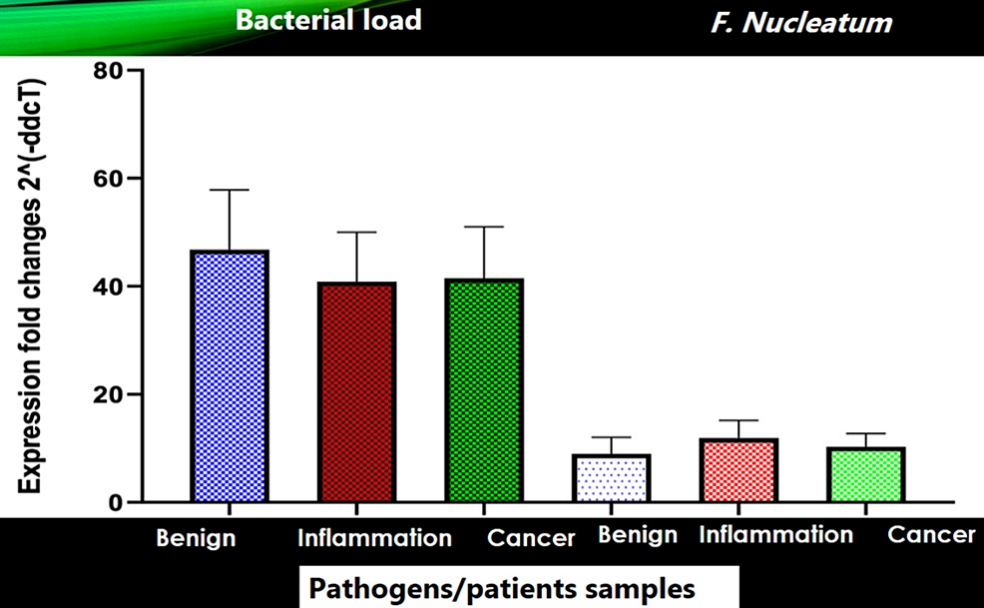
Relative expression fold changes 2^ (- ΔΔCT) of bacterial load and F. nucleatum of all three areas, i.e., BPH, inflammation, and cancer BPH: benign prostatic hyperplasia

The expression of *F. nucleatum* in BPH samples was statistically significant at a p-value <0.0001. *F. nucleatum* in inflammation was statistically significant at p-value <0.0004, and *F. nucleatum* in cancer was statistically significant at p-value<0.0371. The bacterial load from all three areas was approximately the same; in BPH, it was 46.8-fold high, inflammation, it was 40.9-fold high, and in cancer, it was 41.5-fold high. Only *F. nucleatum* was detected among all tested pathogens in BPH, inflammation, and cancer patients samples (Figures 2-3).

In particular, *F. nucleatum* expression was 9, 11.9, and 10.3-fold higher than the expression of *S. epidermidis* in BPH, inflammation, and cancer, respectively, at p-value <0.05. Moreover, *P. gingivalis, P. intermedia, P. denticola*, and *T. forsythia* showed no or zero (CT value undermined or high) expression in BPH, inflammation, and cancer samples. There was no significant difference in bacterial load (folder change) among the three areas (BPH, inflammation, and cancer; p = 0.9). Similarly, there was no significant difference between *F. nucleatum* (folder change) among the three areas (p = 0.879) as there was no significant difference overall using ANOVA. Moreover, *P. gingivalis* strain ATCC 33277, *T. forsythia* strain ATCC 43037, *T. denticola* strain 35405, *P. intermedia* strain B422, and *C. rectus* strain ATCC 33238 were not significantly present in the given samples.

## Discussion

The bacterial load is identified in BPH, inflammation, and cancer areas of the prostate gland diagnosed with malignancy. Moreover, it was determined the presence of *Fusobacterium nucleatum subsp. fusiform *strain ATCC 51190 in BPH, inflammation, and cancer areas. This study identifies the periodontal pathogen *F. nucleatum *in prostate glands diagnosed with adenocarcinoma. However, other periodontal pathogens investigated were not identified. Interestingly, in the study by Estemalik et al., the presence of *P. gingivalis* and *T. denticola* DNA was determined in both the prostatic secretion and dental plaque of the same individual diagnosed with periodontal and prostatic disease [11]. In the present study, the specimen sample was archival prostate tissue, while in the study by Estemalik et al., the specimen sample was prostatic secretion and dental plaque samples from the same individual. In the study by Estemalik et al., the patients included were diagnosed with chronic prostatitis and benign prostatic hyperplasia (BPH), while in the present study, the archival human prostate gland tissue specimens were obtained by the radical prostatectomy from the individuals diagnosed with cancer.

*F. nucleatum* has been identified in colorectal cancer (CRC) tissues [13]. Castellarin et al. identified an overabundance of the *F. nucleatum* in frozen colorectal tumor specimens by quantitative PCR analysis [14]. Furthermore, *F. nucleatum* was reported in individuals with CRC, supporting the concept that *F. nucleatum* could initiate colorectal carcinogenesis [15]. The unique virulence factors of *F. nucleatum* are the presence of adhesion molecules of FadA, endotoxin lipopolysaccharide (LPS), and Fap2. Different pathways were proposed on how *F. nucleatum* creates chronic inflammation in the systemic circulation and carcinogenesis [16]. One of the pathways is the virulence factor FadA of *F. nucleatum* which facilitates adhesion and invasion of the host epithelial and endothelial cells by attaching the cadherin receptor molecule located on the former and later cells. Being one of the cadherin family and a cell-cell junction molecule, vascular endothelial (VE)-cadherin is considered the endothelial receptor for FadA. The binding of FadA to VE-cadherin vessels leads to changing the location of the VE-cadherin to be far from the cell-cell junctions, which increases the permeability of the endothelial cells. Thus, bacteria would pass through the permeable junctions through the endothelium. It is one of the mechanisms by which bacteria disseminate to different body systems to colonize and pass through the placental and blood-brain barriers [17].

The binding of FadA to VE-cadherin leads to the synthesis of a pro-inflammatory cytokine such as IL-6, IL-8, IL-10, IL-18, and TNF-α, creating an environment of chronic inflammation that accelerates the progression of the carcinogenesis [16]. In pathway two, when the virulence factor FadA receptors are present on the vascular endothelial cadherin (E-cadherin), they activate the b-catenin signaling pathway as they bind together, which initiates inflammatory responses, thus boosting the genes of the transcription factors nuclear factor-kB (NF-kB) and the parts of the Wnt pathway. This will advance the proliferation of cell CRC [16]. In pathway three, the Fap2 of *F. nucleatum* interacts with the TIGIT, an inhibitory receptor that leads to the lymphocyte's apoptosis and creates a tumor immunosuppressive environment that further progresses the colorectal tumor [16].

It is unclear whether these periodontal pathogens play a role in these pathologic changes in the prostate. Assessment of such interactions may be worthy of additional future studies. The present study has limitations; determining the presence of periodontal pathogens in the dental plaque samples from the same patients would be desirable, although it was not available for this first study. Additional studies with a larger sample size and adequate control groups, i.e., the healthy prostate gland, are warranted to understand further the role of periodontal pathogens present in the prostatic tissue. We do not have evidence of whether the *F. nucleatum* found in the prostatic tissue of the individual originated from the oral cavity; hence, we do not have evidence on the role of* F. nucleatum* in pathological prostate changes. Well‐controlled longitudinal studies with a larger sample size are warranted to clarify the relationship between periodontal pathogens and/or periodontal disease and prostate disease.

## Conclusions

The chronic inflammation mediated by periodontal pathogens might be associated with prostate diseases. Our study demonstrated that *F. nucleatum *subsp. fusiform strain ATCC 51190 is identified in the prostate that harbor cancer, chronic inflammation, and BPH. However, the *P. gingivalis*, *P. intermedia*, *P. denticola*, *T. forsythia*, and *C. rectus* are not identified. *F. nucleatum* might be associated with the malignancy of the prostate gland. Therefore, *F. nucleatum* may be the link between periodontal diseases and prostate diseases.
